# Supplementary material for: Identifying Key Features Associated with Excessive Fructose Intake: A Machine Learning Analysis of a Mexican Cohort
Source: Nutrients. 2025 Nov 20;17(22):3623. doi: 10.3390/nu17223623 (PMC12655771; doi:10.3390/nu17223623)
Supplement: Supplementary file 1 [file nutrients-17-03623-s001.zip › nutrients-3895934-supplementary.pdf]

nutrients

MDPI

Article

Identifying Key Features Associated with Excessive Fructose Intake: A Machine Learning Analysis of a Mexican Cohort

Guadalupe Gutiérrez-Esparza [1,2,*,†], Mireya Martínez-García [3,4,†], María del Carmen González Salazar [5], Luis M. Amezcua-Guerra [3], Malinalli Brianza-Padilla [3], Tania Ramírez-delReal [1,6] and Enrique Hernández-Lemus [7,*]

1 "Researcher for Mexico" Program, Secretaría de Ciencia, Humanidades, Tecnología e Innovación (SECIHTI), Mexico City, 03940, Mexico
2 Diagnostic and Treatment Services, Instituto Nacional de Cardiología Ignacio Chávez, Mexico City 14080, Mexico
3 Department of Immunology, Instituto Nacional de Cardiología Ignacio Chávez, Mexico City, 14080 Mexico; mireya.martinez@cardiologia.org.mx (M.M.-G.); lmamezcuag@gmail.com (L.M.A.-G.); maly.brianz@gmail.com (M.B.-P.)
4 Dental Public Health Department, Division of Graduate Studies and Research, School of Dentistry, Universidad Nacional Autónoma de México, Mexico City 04510, Mexico
5 Department of Nutrition, Ambulatory Care Services, Instituto Nacional de Cardiología Ignacio Chávez, Mexico City 14080, Mexico; telesforo_13@yahoo.com.mx
6 Centro de Investigación en Ciencias de Información Geoespacial, Aguascalientes 20313, Mexico
7 Computational Genomics Division, Instituto Nacional de Medicina Genómica, Mexico City 14610, Mexico
* Correspondence: ggutierreze@secihti.mx (G.G.-E.); ehernandez@inmegen.gob.mx (E.H.-L.)

† These authors contributed equally to this work.

1. Supplementary Methods

This supplementary document provides a detailed account of the statistical and machine learning methods used in the study. The main manuscript outlines our efforts to identify clinical, nutritional, and behavioral features associated with high fructose intake in a healthy adult Mexican population. Here, we expand on the technical aspects of the computational analyses that supported our findings.

Specifically, this document includes descriptions of the algorithms and performance metrics applied across the different stages of our analysis pipeline. These include the implementation of ensemble classifiers (XGBoost, Random Forest, Histogram-Based Gradient Boosting), unsupervised clustering (K-means), logistic regression modeling, and dimensionality reduction via Principal Component Analysis (PCA). Additionally, we explain the use of SHAP values for model interpretability and provide a summary of clustering validity indices such as silhouette score, Davies-Bouldin index, and Calinski-Harabasz score.

A comprehensive list of all study variables included in the dataset (formerly Table 1 in the main text) is also provided in this supplementary material.

By detailing these analytical procedures, we aim to enhance the transparency and reproducibility of our study, as well as to support readers interested in applying.

Table S1. Overview of the variables included in the descriptive and analytical phases of the study, including their labels, definitions, and data types (C: continuous, D: discrete).

Variable	Description	Type
**Clinical Measurements**		

Variable	Description	Type
Sex	Participant's sex (Male/Female)	D
Height	Height (cm)	C
WC	Waist circumference (cm)	C
Age	Age (years)	C
Weight	Weight (kg)	C
BMI	Body Mass Index (kg/m^2)	C
SBP	Systolic Blood Pressure (mmHg)	C
DBP	Diastolic Blood Pressure (mmHg)	C
**Behavioral Variables**		
LowMets	Low metabolic activity (level)	D
MediumMets	Medium metabolic activity (level)	D
HighAnxState	High anxiety (State)	D
MediumAnxState	Medium anxiety (State)	D
HighAnxRas	High anxiety (Trait)	D
MediumAnxRas	Medium anxiety (Trait)	D
SleepAlt	Sleep alterations (0 – 100)	C
Snoring	Snoring frequency (0 – 100)	C
SleepBR	Sleep breathing risk (0 – 100)	C
SlAdeq	Sleep adequacy (0 – 100)	C
Drowsy	Excessive Daytime Sleepiness (0 – 100)	C
SleepQual	Overall sleep quality (0 – 16)	C
Smoked	Ever smoked (0 = No, 1 = Yes)	D
CurrSmoker	Currently smoking (0 = No, 1 = Yes)	D
DailySmoker	Daily smoker (0 = No, 1 = Yes)	D
ExSmoker	Ex-smoker (0 = No, 1 = Yes)	D
PassSmoker	Passive smoker (0 = No, 1 = Yes)	D
Alcohol	Alcohol consumption (0 = No, 1 = Yes)	D
EnergyDrink	Energy drink consumption (0 = No, 1 = Yes)	C
**Biochemical Parameters**		
URIC	Uric acid (mg/dL)	C
CREA	Creatinine (mg/dL)	C
GLU	Glucose (mg/dL)	C
IAT	Atherogenic index (1-6)	C
CHOL	Total cholesterol (mg/dL)	C
TRIG	Triglycerides (mg/dL)	C
NA	Sodium (mmol/L)	C
**Dietary Intake**		
NatJuicesFruct	Fructose from natural juices (g/day)	C
FruitsFruct	Fructose from fruits (g/day)	C
CerealsFruct	Fructose from cereals (g/day) (g/day)	C
CandiesFruct	Fructose from candies (g/day)	C
SodaFruct	Fructose from soda (g/day)	C
FructNat	Natural fructose (g/day)	C
Added fructose	Added fructose (g/day)	C
VitD	Vitamin D (international units)	C
Protein	Total protein (g/day)	C
ProteinAnimal	Animal protein (g/day)	C
Carbohydrates	Total carbohydrates (g/day)	C
Sucrose	Sucrose (g/day)	C

Variable	Description	Type
Lactose	Lactose (g/day)	C
Maltose	Maltose (g/day)	C
Glucose	Glucose (g/day)	C
Fiber	Total fiber (g/day)	C
Sodium	Sodium (mg/day)	C
Cholesterol	Cholesterol (g/day)	C
FatSaturated	Saturated fat (g/day)	C
FatMonounsaturated	Monounsaturated fat (g/day)	C
FatPolyunsaturated	Polyunsaturated fat (g/day)	C
FatAnimal	Animal fat (g/day)	C
FatVegetal	Vegetable fat (g/day)	C
FructoseTotal	Total fructose (g/day)	C

1.1. XGBoost Algorithm

XGBoost (Extreme Gradient Boosting) is a decision tree-based ensemble algorithm that combines multiple weak learners to create a strong predictive model. It minimizes the following objective function:

$$\mathcal{L}(\theta) = \sum_{i=1}^{n} l(y_i, \hat{y}_i) + \sum_{t=1}^{T} \Omega(f_t) \tag{S1}$$

where l is a differentiable loss function, and $\Omega(f_t)$ is a regularization term that penalizes the complexity of the model.

1.2. SHAP Values for Model Interpretability

SHAP (SHapley Additive exPlanations) is a model-agnostic technique to interpret predictions. Based on cooperative game theory, the Shapley value for feature i is defined as:

$$\phi_i = \sum_{S \subseteq F \setminus \{i\}} \frac{|S|!(|F| - |S| - 1)!}{|F|!} [f(S \cup \{i\}) - f(S)] \tag{S2}$$

where F is the set of all features, and $f(S)$ is the model's output with subset S.

1.3. K-Means Clustering

The K-means partition the data into K clusters by minimizing variance within the cluster. The objective function is:

$$J = \sum_{i=1}^{K} \sum_{x_j \in C_i} \|x_j - \mu_i\|^2 \tag{S3}$$

where μ_i is the centroid of cluster i, and x_j are the data points assigned to it.

1.4. Random Forest Classifier

Random Forest is an ensemble learning method which constructs a multitude of decision trees during training. It reduces variance and avoids overfitting by aggregating the predictions of multiple decorrelated trees.

Each tree is trained on a bootstrap sample of the data, and at each node a random subset of features is selected for splitting. The final prediction is made by majority vote (in classification) or averaging (in regression).

The mathematical prediction for classification is:

$$\hat{y} = \text{mode}(h_t(x))_{t=1}^{T} \tag{S4}$$

Where:

- $\hat{y}$ is the final predicted label.
- T is the total number of trees in the forest.
- $h_t(x)$ is the prediction made by the t-th tree.

The feature importance in Random Forest is commonly computed based on the Gini importance (mean decrease in impurity) across all trees.

1.5. Histogram-Based Gradient Boosting (HistGradientBoosting)

HistGradientBoosting is a variant of traditional gradient boosting that discretizes continuous variables into histograms, enabling more efficient training by reducing computational complexity and memory usage.

This approach follows the gradient boosting paradigm, where trees are built sequentially to fit the pseudo-residuals of the loss function:

$$F_m(x) = F_{m-1}(x) + \nu \cdot h_m(x) \tag{S5}$$

Where:

- $F_m(x)$ is the prediction after the m-th iteration.
- $F_{m-1}(x)$ is the prediction at the previous iteration.
- $h_m(x)$ is the m-th base learner trained on the negative gradient.
- ν is the learning rate (typically between 0.01 and 0.3).

HistGradientBoosting uses histogram binning and early stopping to optimize training efficiency. Feature importance is measured using the total gain of splits per feature.

1.6. Principal Component Analysis (PCA)

Principal Component Analysis (PCA)

PCA is a dimensionality reduction technique that transforms the original variables into a set of orthogonal components that maximize variance. Given a centered data matrix $\mathbf{X} \in \mathbb{R}^{n \times p}$, PCA solves the eigenvalue problem:

$$\mathbf{S}\mathbf{v}_i = \lambda_i \mathbf{v}_i, \tag{S6}$$

where $\mathbf{S} = \frac{1}{n-1}\mathbf{X}^{\top}\mathbf{X}$ is the covariance matrix, $\mathbf{v}_i$ are the eigenvectors, and λ_i are the eigenvalues.

The reduced representation is:

$$\mathbf{Z} = \mathbf{X}\mathbf{V}_k, \tag{S7}$$

where $\mathbf{V}_k$ contains the top k eigenvectors. In our study, $k = 2$ was selected to retain the highest variance.

1.7. ANOVA and Eta Squared

One-way ANOVA was used to compare continuous variables across clusters. The effect size (η^2) was calculated as:

$$\eta^2 = \frac{SS_{between}}{SS_{total}} \tag{S8}$$

where $SS_{between}$ is the sum of squares between groups and SS_{total} is the total sum of squares.

1.8. Logistic Regression Classifier

Logistic regression estimates the probability that an observation belongs to a class using the sigmoid function:

$$P(y=1|X) = \frac{1}{1+e^{-(\beta_0+\beta_1 x_1+\ldots+\beta_p x_p)}} \tag{S9}$$

Where:

- $P(y=1|X)$: Probability of positive class.
- β_0: Intercept.
- $\beta_1, \ldots, \beta_p$: Coefficients.
- $x_1, \ldots, x_p$: Feature values.

The threshold 0.5 was used for classification.

1.9. Evaluation Metrics

To assess classification performance, the following metrics were calculated:
Balanced accuracy: Average of sensitivity and specificity.
Balanced Accuracy:

$$\text{Balanced Accuracy} = \frac{1}{2}\left(\frac{TP}{TP+FN} + \frac{TN}{TN+FP}\right) \tag{S10}$$

Sensitivity (Recall):

$$\text{Sensitivity} = \frac{TP}{TP+FN} \tag{S11}$$

Specificity:

$$\text{Specificity} = \frac{TN}{TN+FP} \tag{S12}$$

F1-score: Harmonic mean of precision and recall.

$$F1 = 2 \cdot \frac{\text{Precision} \cdot \text{Recall}}{\text{Precision} + \text{Recall}} \tag{S13}$$

Area Under the Curve (AUC) was calculated as the integral of the ROC curve, which plots sensitivity against 1-specificity across all decision thresholds.

1.10. Clustering Metrics

The silhouette score quantifies the degree to which an individual observation belongs to its assigned cluster in contrast to other clusters.

$$s(i) = \frac{b(i) - a(i)}{\max\{a(i), b(i)\}}$$

where $a(i)$ is the mean intra cluster distance and $b(i)$ is the mean nearest cluster distance. The score ranges from -1 to 1, where higher values indicate better defined clusters.

The Davies-Bouldin index measures the average similarity between each cluster and its most similar one, based on the ratio of within-cluster distances to between-cluster distances:

$$DB = \frac{1}{n}\sum_{i=1}^{n} \max_{j \neq i}\left(\frac{\sigma_i + \sigma_j}{d_{ij}}\right)$$

where σ_i and σ_j are the distances within the cluster and d_{ij} is the distance between the centroids of the clusters. Lower values indicate better clustering.

The Calinski-Harabasz score, also known as the variance ratio criterion, evaluates the ratio of between-cluster dispersion to within-cluster dispersion:

$$CH = \frac{\mathrm{Tr}(B_k)}{\mathrm{Tr}(W_k)} \cdot \frac{N-k}{k-1}$$

where $\mathrm{Tr}(B\_k)$ and $\mathrm{Tr}(W\_k)$ are the traces of the dispersion matrices between clusters and within clusters, respectively, N is the number of samples, and k is the number of clusters. Higher values indicate more distinct and well-separated clusters.

Table S2. Top five variables contributing most strongly to each principal component based on absolute loading values.

Variable	Component	Loading Value
`Protein`	PCA1	0.3063
`FatSaturated`	PCA1	0.3011
`Sodium`	PCA1	0.2873
`FatMonounsaturated`	PCA1	0.2861
`Carbohydrates`	PCA1	0.2799
`Weight`	PCA2	0.3799
`WC`	PCA2	0.3646
`BMI`	PCA2	0.3182
`SBP`	PCA2	0.2964
`URIC`	PCA2	0.2929

Protein: Total protein (g/day); FatSaturated: Saturated fat (g/day); Sodium: Sodium (g/day); FatMonounsaturated: Monounsaturated fat (g/day); Carbohydrates: Total carbohydrate (g/day); WC: Waist circumference (cm); BMI: Body Mass Index; SBP: Systolic Blood Pressure (mmHg); URIC: Uric acid (mg/dL).

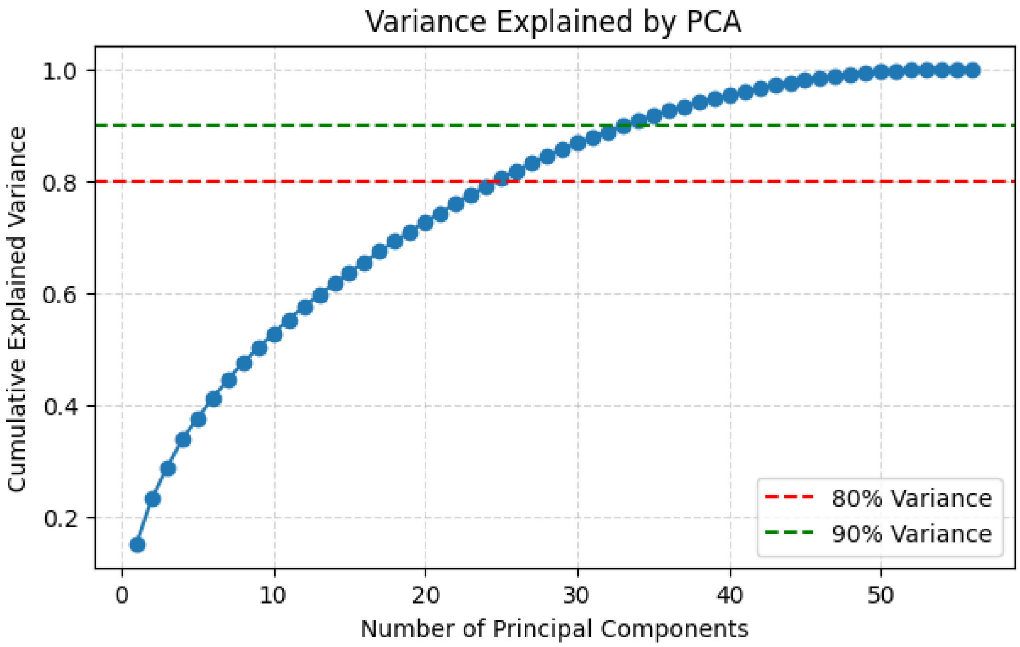

Figure S1. Cumulative explained variance of the principal components.
Note: The first two components together explain less than 25% of the total variance, indicating that the dataset is highly complex and multidimensional. Approximately 26 components are required to explain 80% of the total variance, while about 38 components are needed to reach 90%. Due to this low variance concentration in the leading components, PCA was not used as a dimensionality reduction technique for clustering. Instead, clustering analysis was performed directly on the full set of standardized variables to avoid information loss.

Supplementary Material

Table S3. Skewness coefficients for dietary variables. Most variables show moderate or low skewness ($|\text{skew}| < 2$), supporting the decision to retain the original scale.

Variable	Skewness	| Skew |
NatJuicesFruct	33.32	33.32
FrcutNat	17.89	17.89
FruitsFruct	16.25	16.25
CandiesFruct	2.68	2.68
VitD	2.64	2.64
Cholesterol	2.59	2.59
CerealsFruct	2.26	2.26
Glucose	2.25	2.25
SodaFruct	2.25	2.25
Lactose	1.99	1.99
FatMonounsaturated	1.97	1.97
FatVegetal	1.81	1.81
Sodium	1.75	1.75
FatAnimal	1.68	1.68
FatSaturated	1.60	1.60
FatPolyunsaturated	1.59	1.59
Maltose	1.37	1.37
Protein	1.34	1.34
FructAdded	1.29	1.29
ProteinAnimal	1.25	1.25
Sucrose	1.07	1.07
Fiber	1.05	1.05
Carbohydrates	0.84	0.84
